# Interrupting sitting acutely attenuates cardiometabolic risk markers in South Asian adults living with overweight and obesity

**DOI:** 10.1007/s00421-023-05345-7

**Published:** 2023-11-11

**Authors:** Kamalesh Chandra Dey, Julia K. Zakrzewski-Fruer, Lindsey R. Smith, Rebecca L. Jones, Daniel P. Bailey

**Affiliations:** 1 Institute for Sport and Physical Activity Research, School of Sport Science and Physical Activity, University of Bedfordshire, Bedford, UK; 2 Preventive Neurology Unit, Wolfson Institute of Population Health, Barts and the London School of Medicine and Dentistry, Queen Mary University of London, London, UK; 3 Health Advancement Research Team (HART), School of Sport and Exercise Science, University of Lincoln, Lincoln, UK; 4 Division of Sport, Health and Exercise Sciences, Department of Life Sciences, Brunel University London, Uxbridge, UK; 5 Centre for Physical Activity in Health and Disease, Brunel University London, Uxbridge, UK

**Keywords:** Obesity, Sedentary behaviour, Cardiometabolic health, Glucose, Metabolic load index, South Asian

## Abstract

**Purpose:**

This study examined the acute effects of interrupting sitting with light-intensity walking on postprandial cardiometabolic risk markers in South Asian adults.

**Methods:**

South Asians with overweight/obesity (*n* = 19; body mass index [BMI] > 23 kg·m^−2^) and normal-weight (*n* = 8; BMI 18.0–22.9 kg·m^−2^) aged 48.8 ± 5.6 years completed two, 5-h conditions: (1) prolonged sitting (SIT), and (2) interrupted sitting with 5-min bouts of light-intensity walking every 30-min (INT-SIT). Blood samples and resting expired air samples were collected throughout each condition. Statistical analyses were completed using linear mixed models.

**Results:**

In participants with overweight/obesity, postprandial glucose, triglycerides (TAG) and metabolic load index (MLI) over time were lower, whereas resting substrate utilisation and resting energy expenditure (REE) were higher, in INT-SIT than SIT (all *p* ≤ 0.05). Compared with SIT (0.18 [95% CI 0.13, 0.22] kcal^.^min^−1^), INT-SIT (0.23 [95% CI 0.18, 0.27] kcal^.^min^−1^) increased postprandial REE iAUC in participants with overweight/obesity (*p* = 0.04, *d* = 0.51). Postprandial TAG concentrations over time were lower in INT-SIT versus SIT (*p* = 0.01, *d* = 30) in normal-weight participants, with no differences in any other outcomes for this sample group.

**Conclusion:**

These findings suggest that interrupting sitting with 5-min bouts of light walking every 30-min acutely attenuates cardiometabolic risk markers among South Asians living with overweight/obesity, whereas limited effects may be seen in individuals with normal-weight.

**Supplementary Information:**

The online version contains supplementary material available at 10.1007/s00421-023-05345-7.

## Introduction

South Asians are the indigenous population originating from the Indian subcontinent, including Bangladesh, India, Pakistan, Sri-Lanka, and Nepal (Jalal et al. 2019). Individuals of this ethnic background represent approximately 25% of the world’s population (The World Bank Group 2021). South Asians have a significantly increased risk (up to six times) of developing cardiometabolic diseases, such as cardiovascular disease (CVD) and Type 2 diabetes, compared with other ethnic groups (Hanif and Susarla 2018; Misra and Khurana 2011). This may be in part due to having a thin outside, fat inside phenotype; thus, South Asians have excess internal body fat (e.g., higher abdominal, visceral, and hepatic fat), lower lean muscle mass, and greater accumulation of fat in ectopic sites including liver and skeletal muscle, compared to other ethnic groups (Bays et al. 2022; Misra and Khurana 2011). In attempts to account for this distinct phenotype, lower body mass index (BMI) and waist circumference cut-off points have been proposed for South Asians (Misra and Khurana 2011). Obesity, and in particular abdominal fatness, is a predisposing factor in the development of cardiometabolic disease, possibly due to the presence of insulin resistance (Gray et al. 2011). For instance, type 2 diabetes is reported to be five times more prevalent in individuals with obesity than normal-weight adults (Abdullah et al. 2010). Thus, South Asians living with overweight/obesity are considered a high-risk group that would benefit from interventions to reduce the risk of cardiometabolic disease.

Overweight and obesity is associated with lower physical activity and higher sedentary time in the general population (Gibbs et al. 2017; Van Dyck et al. 2015). Increased physical activity engagement in individuals with overweight and obesity is related to self-perceived good health, self-efficacy, social support and exercise enjoyment (Curran et al. 2023). There is some evidence that being an office worker, severity of obesity and number of comorbidities is related to greater sedentary time (Curran et al. 2023). Higher sedentary time is detrimentally associated with cardiometabolic risk markers (such as fasting glucose and triglycerides [TAG]) and incidence of type 2 diabetes, CVD and CVD mortality (Bailey et al. 2019; Healy et al. 2011; Patterson et al. 2018). Increased postprandial hyperglycaemia and hyperlipidaemia are strong predictors of cardiometabolic disease and may represent a suitable intervention target for managing cardiometabolic health (Einarson et al. 2011; O’Keefe and Bell 2007). Interrupting prolonged sitting with 1-min 40-s to 5-min bouts of light or moderate-intensity walking every 20 to 30-min significantly attenuated postprandial cardiometabolic risk markers (e.g., glucose and insulin) in Caucasian participants who are normal-weight or living with overweight and obesity (Champion et al. 2018; Dunstan et al. 2012; Henson et al. 2016; Larsen et al. 2015; Peddie et al. 2013). Interrupting prolonged sitting with 5-min bouts of light walking also attenuated postprandial insulin in older South Asians (aged 65–79 years) with mixed weight status (Yates et al. 2020). Fat oxidation and resting energy expenditure (REE) were also significantly higher in response to interrupting sitting with light or moderate-intensity walking for 1 to 2-min every 30-min, chair squats or intermittent standing every 20-min, compared with prolonged sitting in Caucasians living with normal-weight or overweight/obesity (Hawari et al. 2019, 2016; Larsen et al. 2017; Peddie et al. 2013). Thus, interrupting sitting has acute beneficial cardiometabolic and substrate utilisation effects.

The benefits of interrupting sitting appear to be more pronounced in participants with overweight and obesity than those with normal-weight. This could be due to a larger scope for improvement as well as physical activity being a more stressful stimulus in less fit populations (Bell et al. 2023). South Asians spend a large proportion of their time being sedentary, particularly when measured using device-based methods (~ 9 h/day) (Dey et al. 2021). This could place them at increased cardiometabolic disease risk, especially if they are overweight or obese (Ahmad et al. 2017). Yet, there is limited evidence examining the effects of interrupting sitting on cardiometabolic health in South Asian adults of differing weight status. Such research is important to inform specific target groups that may benefit from this type of intervention.

This study aimed to determine the effects of interrupting sitting with light walking on postprandial cardiometabolic risk markers (primary outcome) and resting metabolic rate (i.e., resting substrate utilisation and energy expenditure; secondary outcomes) in South Asian adults living with normal-weight and overweight/obesity. It was hypothesised that interrupting sitting would attenuate postprandial glucose in South Asian adults, but this effect would be more pronounced in participants with overweight/obesity.

## Methods

### Study overview

This two-condition randomised crossover study was conducted according to the Declaration of Helsinki principles and approved by the University of Bedfordshire Institute for Sport and Physical Activity Research Ethics Committee (2019ISPAR003). Participants provided written informed consent prior to taking part in any study procedures. The study was conducted and reported in line with the Consolidated Standards of Reporting Trials (CONSORT) guidance (Moher et al. 2012); see checklist in Supplementary Material S1. The trial was registered with clinicaltrials.gov/ (NCT03898206). Experimental condition order was randomised using an online computer-generated randomisation method (https://www.randomizer.org/). Following a preliminary testing visit, participants completed two experimental conditions separated by ≥ 3 to 28 days to eliminate potential carryover effects (Mikines et al. 1988). Female participants were tested between 1 and 10 days into their follicular phase to minimise the influence of hormonal fluctuations on glucose metabolism (Pulido and Salazar 1999). The study was interrupted by the COVID-19 pandemic, affecting the ability to continue data collection; the associated impact on the study protocol is described below. All testing procedures took place at the University of Bedfordshire Sport and Exercise Science Laboratories.

### Participants

South Asian adults aged 18 to 75 years who were normal-weight (BMI 18.0–22.90 kg^.^m^−2^) or overweight/obese (BMI > 23 kg^.^m^−2^) were eligible to take part. These BMI thresholds were used in line with recommendations for South Asian populations (Bays et al. 2022). Body mass index was calculated as body mass (kg) ÷ height (m)^2^. ‘South Asian’ was defined as anyone identifying themselves as South Asian or British South Asian (Bays et al. 2022). Exclusion criteria were self-reported CVD, diabetes, any known blood borne disease, pregnancy, recent or current smoker, allergies to the test meals, other known health issues (e.g., neurological disorders), or any injuries that might limit the ability to perform light walking. Participants were recruited from the local community (Luton and Bedford, UK) using adverts through social media posts (e.g., Facebook and Twitter) and distribution of flyers.

#### Sample size calculations

This study was originally designed to investigate the interaction between weight status and interrupted sitting on cardiometabolic outcomes*.* The primary outcome was postprandial glucose iAUC. An *a-priori* sample size was calculated to provide sufficient power to detect a weight status x experimental condition interaction, which resulted in an estimated 52 participants being required (26 in each the normal-weight and overweight/obese groups). Unfortunately, data collection was suspended due to the COVID-19 pandemic, which meant that it was no longer possible to complete data collection for the intended 52 participants within the time constraints of the study. Thus, sample size estimations were re-visited in an attempt to use the available data in the most meaningful way. Rather than examining the weight status x experimental condition interaction, analyses comparing experimental conditions were conducted separately for normal-weight and participants with overweight/obesity, with between-group comparisons interpreted descriptively in the discussion rather than being determined statistically (Gondim et al. 2015).

To detect statistical significance between conditions in each group separately, a total of nine normal-weight and 12 participants with overweight/obesity were required. These calculations were based on a mean effect size of *d* = 1.12 for normal-weight and *d* = 0.89 for participants with overweight/obesity from previous research (Bailey and Locke 2015; Dunstan et al. 2012; Henson et al. 2016; Peddie et al. 2013; Yates et al. 2020) to achieve 80% power with an alpha level of 5%. A total of 12 normal-weight and 16 participants with overweight/obesity, allowing for a 20% dropout, was the revised target sample size. Sample size calculations were performed using G* power (version 3.0.10; Germany).

### Preliminary measures

Height (cm) was measured to the nearest 0.1 cm using a stadiometer (Harpenden 98.602, Crymych, UK). Body mass (kg) was measured to the nearest 0.1 kg, and the percentage of body fat (BF %) was estimated after fasting for 4 h, using the Tanita BC-418 Segmental Body Composition Analyzer (Tanita Corp., Tokyo, Japan). Waist circumference (cm) was measured at minimal inspiration to the nearest 0.1 cm (Lohman et al. 1988). Resting blood pressure (BP; mmHg) and heart rate (HR; beats^.^min^−1^) were measured using an automatic device (Omron M5-I; Omron Matsusaka Co. Ltd., Matsusaka, Japan). Participants were familiarised with the Borg Rating of Perceived Exertion (RPE) scale (Borg 1982) and the Woodway motorised treadmill (Woodway PPS55Med-i, GmbH, Germany). An exercise protocol to determine the walking speed for each participant in the relevant experimental condition began at a speed of 1.2 km^.^h^−1^ and increased by 0.5 km^.^h^−1^ every 2-min until an RPE of 9 (very light) was reached. This speed was recorded for each participant (Bailey and Locke 2015).

### Experimental protocol

**Fig. 1:**
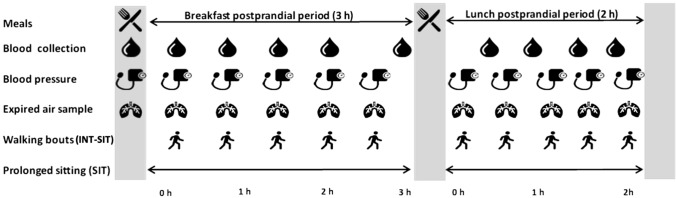
Schematic of the experimental protocol

Participants attended two separate laboratory visits at 08:30 am in the fasted state (Henson et al. 2016). They were asked to refrain from food and drink containing alcohol and caffeine for 24-h before and to avoid moderate-to-vigorous exercise 48-h before each condition to exclude possible acute influences on insulin sensitivity (Mikines et al. 1988). Participants were asked to weigh and record all food and beverages consumed for 24-h before their first experimental condition and then consume the same volume of food and beverage at the exact times in the 24-h before their second experimental condition. This was to minimise the influence of chronobiological aspects of dietary intake (e.g., timing, and frequency) and macronutrient intake on cardiometabolic risk marker responses (Ekmekcioglu and Touitou 2011). Participants were instructed to travel by car and park as close as possible to the laboratories to minimise physical activity in the hours before each condition. Upon arrival, participants sat for 5-min and resting BP and HR were measured. Afterwards, resting expired air was collected continuously for 5-min using a Metalyzer 3B (Cortex Biophysik, Leipzig, Germany). A fasting blood sample was then taken immediately before consuming a standardised breakfast. The 5-h experimental condition began immediately after the breakfast was consumed. The conditions were as follows, as illustrated in Fig. 1:

1. SIT: Participants remained seated at a desk for 5-h and were instructed to reduce excessive movement.
2. INT-SIT: Participants interrupted their sitting every 30-min with walking on a motorised treadmill at a light-intensity for 5-min. Participants started the walking breaks at 30, 60, 90, 120, 150 and 180-min into the breakfast postprandial period and 30, 60 and 90-min into the lunch postprandial period. The walking breaks were undertaken on nine occasions, providing a total of 45-min of light walking.

A standardised lunch meal was provided at 3 h into each condition. Participants were permitted to read books, newspapers, and magazines, or work on a laptop/computer throughout the conditions. When participants needed to use the toilet, they were transported in a wheelchair to avoid physical activity.

### Test meals

The standardised breakfast and lunch meals both provided 25% of the estimated daily energy requirements for each participant (Mattes and Campbell 2009). Energy requirements were estimated based on each participant’s age and body mass using Mifflin equations (Mifflin et al. 1990). Breakfast consisted of cornflakes, whole milk, jam sandwich and orange juice that comprised 58% carbohydrate, 28% fat and 13% protein (Champion et al. 2018). Lunch consisted of a chicken (non-halal or halal) or cheese sandwich, salted crisps, chocolate and Lucozade original drink that comprised 58% carbohydrate, 28% fat and 13% protein (Champion et al. 2018). The glycaemic index (GI) of the breakfast and lunch was 73 and 74, respectively; values were obtained from the International Tables of GI and Glycaemic Load Values 2008 (Atkinson et al. 2008). The macronutrient content and GI of the meals were intended to provide a postprandial stimulus that would be sensitive to breaks in sitting (Augustin et al. 2015). Participants were asked to consume each meal within 15-min and replicate the consumption times from the first condition in the second condition (Henson et al. 2016). Water was provided ad libitum during the first condition, and the total volume consumed was recorded. Participants were asked to replicate this amount during the second condition (Henson et al. 2016).

### Blood collection and biochemistry

Capillary finger prick blood samples were collected in a fasted state at baseline followed by subsequent samples at 25, 55, 85, 115, and 175-min during the breakfast postprandial period and at 25, 55, 85, and 115-min during the lunch postprandial period. Whole blood was used to analyse glucose (mmol^.^L^−1^) using the YSI 2300 STAT glucose analyser (YSI Inc., Yellow Springs, OH, USA) and TAG (mmol^.^L^−1^) using the Reflotron®Plus (Roche Diagnostics, Burgess Hill, UK). The remaining blood was centrifuged using a micro-centrifugal machine (Heraeus Pico 17 microcentrifuge, Loughborough, UK) at 2000 × g for 5-min (Bailey and Locke 2015). Plasma was extracted and stored at − 80 °C. Plasma insulin (mU^.^L^−1^) was analysed using an enzyme-linked immunosorbent assay kit (Mercodia AB, Uppsala, Sweden).

### Resting substrate utilisation and energy expenditure

Expired air samples were collected for 5-min after a 10–20 min rest to estimate resting fat oxidation (mg^.^min^−1^), carbohydrate oxidation (mg^.^min^−1^) and REE (kcal^.^min^−1^) using the Metalyzer 3B. These samples were taken in a fasted state at baseline followed by samples at 20, 50, 80, 110, 140, and 170-min during the breakfast postprandial period and 20, 50, 80, and 110-min during the lunch postprandial period.

### Blood pressure and heart rate measurement

Resting BP (mmHg) and HR (beats^.^min^−1^) were measured on the left arm while seated in an upright resting position. At baseline, three readings were taken with the mean from the lowest two measurements being recorded. Subsequent single readings were taken at 15, 45, 75, 105, 135 and 165-min during the breakfast postprandial period and 15, 45, 75 and 105-min during the lunch postprandial period.

### Calculation of outcome variables

The total 300-min (5-h) area under the curve (tAUC), incremental AUC (iAUC) and positive iAUC (p-iAUC; i.e., excluding any data below baseline) were calculated for postprandial blood glucose, TAG, metabolic load index (MLI) and plasma insulin. Total area under the curve and iAUC were calculated for resting fat oxidation, carbohydrate oxidation and REE. Area under the curve was calculated using the trapezoidal rule and p-iAUC was calculated as tAUC above the baseline value (Wolever and Jenkins 1986). The MLI was calculated using the following equation: MLI (mmol^.^L^−1^) = blood glucose (mmol^.^L^−1^) + TAG (mmol^.^L^−1^) (Emerson et al. 2016). Resting substrate utilisation was calculated by indirect calorimetry using stoichiometric equations with the assumption that the contribution of protein to energy expenditure is negligible (Frayn 1983). The following formula was used to calculate mean arterial pressure (MAP), where P is BP (systolic or diastolic) (DeMers and Wachs 2019): MAP = P_Diastolic_ + 1/3 (P_Systolic_ – P_Diastolic_).

### Statistical analysis

Statistical analyses were performed using SPSS version 26.0 (SPSS Inc., NY, USA). Data were tested for normality using Q–Q plots prior to statistical analysis. Linear mixed models were used to determine the main effect of condition (INT-SIT vs. SIT) for the AUC variables and the condition × time (i.e. sample time points during each condition) interaction for all other outcomes. All models were adjusted for condition order and the baseline value of each outcome. Condition, time and covariates were included as fixed factors and participants were a random factor in each model. Two-tailed statistical significance was set at *p* ≤ 0.05. Cohen’s *d* effect sizes were calculated with 0.2, 0.5, and 0.8 indicating a small, medium or large effect, respectively (Cohen 1988). All data are presented as mean (95% confidential interval [CI]) unless stated otherwise.

## Results

Recruitment and data collection of participants took place between April 2019 and March 2020. Participant flow throughout the study is shown in Supplementary Material S2. Following screening, 54 participants were enrolled into the study. Twenty-seven participants were unable to attend the laboratory to complete data collection due to the COVID-19 pandemic. Therefore, 27 (eight normal-weight and 19 with overweight/obesity) datasets were included in the analysis. Descriptive characteristics of the participants are presented in Table 1. The average (SEM) walking bout speed for the normal-weight and overweight/obese group was 2.5 ± 0.2 and 2.6 ± 0.2 km^.^h^−1^, respectively. There were no differences in the baseline variables between the two experimental conditions in either the normal-weight or overweight/obese groups (see Table 2).

**Table 1:** Description of the participants' characteristics

Characteristics	Overweight/obese (*n* = 19)	Normal-weight (*n* = 8)
Ethnic origin		
Bangladeshi (n)	1 (5)	2 (25)
Indian (n)	10 (53)	5 (63)
Nepali (n)	1 (5)	1 (12)
Pakistani (n)	7 (37)	0 (0)
Religion		
Hinduism (n)	5 (26)	8 (100)
Sikhism (n)	8 (42)	0 (0)
Muslim (n)	6 (32)	0 (0)
Employment status		
Employed	18 (95)	8 (100)
Retired	1 (5)	0 (0)
Female	14 (74)	6 (75)
Male	5 (26)	2 (25)
Age (years)	50.4 ± 3.3	47.2 ± 7.8
Body mass (kg)	67.9 ± 1.8	53.9 ± 2.5
Body mass index (kg^.^m^−2^)	26.4 ± 0.6	20.6 ± 0.5
Body fat (%)	34.3 ± 1.7	23.8 ± 2.7
Waist circumference (cm)	92.7 ± 2.3	73.6 ± 3.5

Data presented as mean ± standard error of the mean and *n* (%)

**Table 2:** Cardiometabolic risk marker values for the two experimental conditions

	Prolonged sitting	Interrupted sitting with light walking	*p*-value for the main effect of condition	Cohens’ *d *effect size
**Overweight/obese participants (*n* = 19)**				
Baseline blood glucose (mmol^.^L^−1^)	4.69 (4.56, 4.82)	4.59 (4.46, 4.72)	0.29	0.30
Baseline triglycerides (mmol^.^L^−1^)	1.49 (1.24, 1.73)	1.34 (1.10, 1.58)	0.15	0.31
Baseline insulin (mU^.^L^−1^)	7.21 (4.13, 10.28)	6.79 (3.72, 9.86)	0.59	0.09
Baseline mean arterial pressure (mmHg)	94.42 (88.40, 100.45)	95.35 (89.32, 101.38)	0.58	0.07
Baseline heart rate (beats^.^min^−1^)	68.69 (63.57, 73.81)	70.01 (64.89, 75.13)	0.48	0.12
Blood glucose tAUC (mmol^.^L^−1.^5 h^−1^)	6.37 (6.05, 6.70)	6.17 (5.84, 6.49)	0.09	0.31
Blood glucose iAUC (mmol^.^L^−1.^5 h^−1^)	1.74 (1.41, 2.06)	1.53 (1.20, 1.85)	0.09	0.30
Blood glucose p-iAUC (mmol^.^L^−1.^5 h^−1^)	1.77 (1.46, 2.09)	1.57 (1.26, 1.89)	0.08	0.30
Blood glucose concentration over time^a^ (mmol^.^L^−1^)	6.37 (6.06, 6.68)	6.18 (5.87, 6.49)	**0.02**	0.37
Triglycerides tAUC (mmol^.^L^−1.^5 h^−1^)	2.09 (1.87, 2.31)	2.05 (1.83, 2.26)	0.66	0.10
Triglycerides iAUC (mmol^.^L^−1.^5 h^−1^)	0.67 (0.45, 0.89)	0.63 (0.41, 0.85)	0.66	0.10
Triglycerides p-iAUC (mmol^.^L^−1.^5 h^−1^)	0.68 (0.47, 0.89)	0.65 (0.45, 0.86)	0.80	0.06
Triglycerides concentration over time^a^ (mmol^.^L^−1^)	2.09 (1.87, 2.31)	2.01 (1.79, 2.23)	**0.05**	0.19
Metabolic load index tAUC (mmol^.^L^−1.^5 h^−1^)	8.44 (8.04, 8.84)	8.22 (7.82, 8.62)	0.18	0.26
Metabolic load index iAUC (mmol^.^L^−1.^5 h^−1^)	2.39 (1.98, 2.79)	2.17 (1.76, 2.57)	0.18	0.26
Metabolic load index p-iAUC (mmol^.^L^−1.^5 h^−1^)	2.42 (2.03, 2.81)	2.24 (1.85, 2.63)	0.25	0.23
Metabolic load index over time^a^ (mmol^.^L^−1^)	8.44 (8.06, 8.83)	8.20 (7.81, 8.58)	**0.01**	0.32
Plasma insulin tAUC (mU^.^L^−1.^5 h^−1^)	51.36 (42.68, 60.04)	48.30 (39.61, 56.98)	0.56	0.16
Plasma insulin iAUC (mU^.^L^−1.^5 h^−1^)	44.19 (35.50, 51.87)	41.12 (32.44, 49.80)	0.56	0.16
Plasma insulin p-iAUC (mU^.^L^−1.^5 h^−1^)	44.22 (35.56, 52.88)	41.07 (32.41, 49.74)	0.54	0.17
Plasma insulin concentration over time^a^ (mU^.^L^−1^)	51.91 (44.65, 59.16)	48.82 (41.57, 56.06)	0.31	0.83
Mean arterial pressure (mmHg)	94.22 (92.05, 96.38)	93.33 (91.16, 95.51)	0.43	0.02
Heart rate (beats^.^min^−1^)	69.24 (66.92, 71.56)	69.76 (67.44, 72.08)	0.69	0.15

	Prolonged sitting	Interrupted sitting with light walking	*p*-value for the main effect of condition	Cohens’ *d* effect size
**Normal-weight participants (***n* = **8)**				
Baseline blood glucose (mmol^.^L^−1^)	4.62 (4.18, 5.06)	4.60 (4.15, 5.04)	0.94	0.05
Baseline triglycerides (mmol^.^L^−1^)	1.13 (0.90, 1.37)	1.15(0.91, 1.34)	0.91	0.04
Baseline insulin (mU^.^L^−1^)	5.50 (1.74, 9.26)	4.06 (0.31, 7.82)	0.16	0.33
Baseline mean arterial pressure (mmHg)	86.93 (75.21, 98.65)	88.91 (77.19, 100.63)	0.40	0.18
Baseline heart rate (beats^.^min^−1^)	67.41 (62.04, 72.77)	68.22 (62.85, 73.59)	0.68	0.14
Blood glucose tAUC (mmol^.^L^−1.^5 h^−1^)	6.12 (5.61, 6.62)	6.06 (5.56, 6.56)	0.77	0.09
Blood glucose iAUC (mmol^.^L^−1.^5 h^−1^)	1.46 (1.09, 1.84)	1.43 (1.05, 1.71)	0.88	0.07
Blood glucose p-iAUC (mmol^.^L^−1.^5 h^−1^)	1.46 (1.10, 1.83)	1.46 (1.09, 1.83)	0.99	0.05
Blood glucose concentration over time^a^ (mmol^.^L^−1^)	5.95 (5.41, 6.49)	5.95 (5.41, 6.49)	0.99	0.03
Triglycerides tAUC (mmol^.^L^−1.^5 h^−1^)	1.50 (1.22, 1.79)	1.38 (1.10, 1.67)	0.14	0.35
Triglycerides **i**AUC (mmol^.^L^−1.^5 h^−1^)	0.35 (0.07, 0.64)	0.23 (-0.05, 0.52)	0.14	0.35
Triglycerides p-iAUC (mmol^.^L^−1.^5 h^−1^)	0.37 (0.13, 0.61)	0.33 (0.09, 0.57)	0.69	0.14
Triglycerides concentration over time^a^ (mmol^.^L^−1^)	1.42 (1.08, 1.76)	1.30 (0.96, 1.64)	**0.01**	0.30
Metabolic load index **t**AUC (mmol^.^L^−1.^5 h^−1^)	7.34 (6.52, 8.15)	7.17 (6.36, 7.99)	0.40	0.16
Metabolic load index iAUC (mmol^.^L^−1.^5 h^−1^)	1.76 (1.07, 2.44)	1.60 (0.91, 2.29)	0.43	0.19
Metabolic load index p-iAUC (mmol^.^L^−1.^5 h^−1^)	1.80 (1.17, 2.43)	1.76 (1.13, 2.39)	0.84	0.05
Metabolic load index over time^a^ (mmol.L^−1^)	7.32 (6.52, 8.12)	7.21 (6.41, 8.01)	0.38	0.12
Insulin tAUC (mU^.^L^−1.^5 h^−1^)	30.02 (22.22, 37.83)	32.28 (24.48, 40.08)	0.65	0.22
Insulin iAUC (mU^.^L^−1.^5 h^−1^)	24.69 (16.89, 32.50)	26.95 (19.14, 34.75)	0.65	0.22
Insulin p**-**iAUC (mU^.^L^−1.^5 h^−1^)	24.73 (17.54, 31.92)	26.26 (19.07, 33.45)	0.74	0.16
Plasma insulin concentration over time^a^ (mU^.^L^−1^)	30.55 (22.30, 38.81)	32.68 (24.31, 41.05)	0.44	0.14
Mean arterial pressure (mmHg)	89.17 (85.96, 92.38)	87.24 (84.03, 90.45)	0.32	0.02
Heart rate (beats^.^min^−1^)	67.50 (64.37, 70.62)	67.62 (64.49, 70.74)	0.93	0.11

Data presented as mean (95% confidence interval); tAUC, total area under the curve; iAUC, incremental area under the curve; p-iAUC, positive incremental area under the curve; ^a^values refer to the marginal means for the main effect of condition in the condition by time analyses; Statistically significant (*p* ≤ 0.05) differences highlighted in **bold**

### Cardiometabolic risk markers

**Fig. 2:**
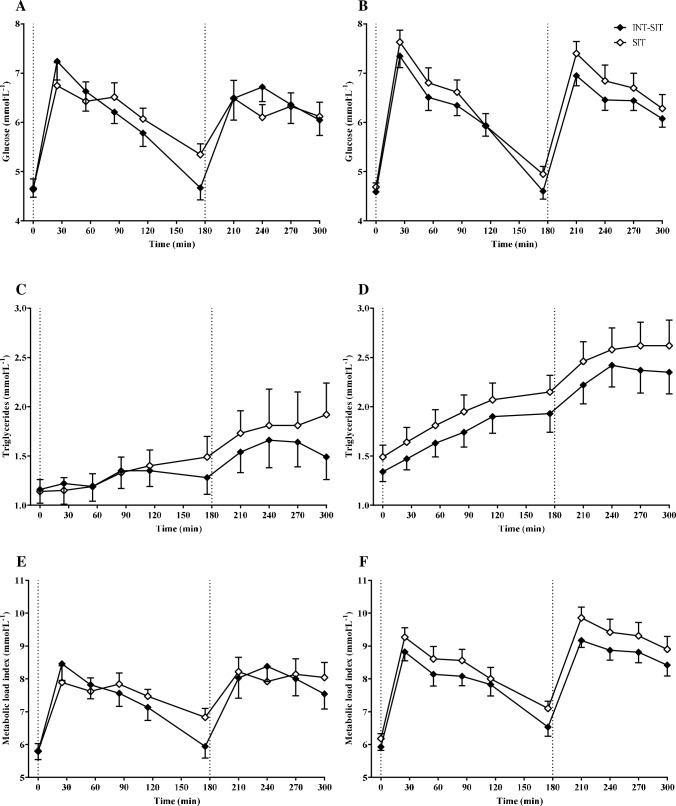
Postprandial blood glucose (**A**), triglycerides (**C**), and metabolic load index (**E**) in normal-weight South Asians and blood glucose (**B**), triglycerides (**D**) and metabolic load index (**F**) in South Asians living with overweight/obesity for the prolonged sitting (SIT) and sitting interrupted with light walking (INT-SIT) conditions. Data are mean and 95% confidence interval. Dotted lines indicate standardised test meals for breakfast and lunch

Postprandial glucose, triglycerides and MLI over time for each condition are shown in Fig. 2. Blood glucose concentrations across the 5-h were lower by -0.20 mmol^.^L^−1^ (95% CI -0.36, -0.04) in INT-SIT than SIT in the overweight/obese group (see Table 2). There was a trend for lower postprandial glucose iAUC in INT-SIT compared with SIT in the overweight/obese group. Triglycerides and MLI across the 5-h were attenuated in the overweight/obese group in INT-SIT by -0.09 mmol^.^L^−1^ (95% CI -0.17, 0.00) and -0.25 mmol.L^−1^ (95% CI -0.43, -0.06), respectively, compared with SIT (Table 2). Postprandial insulin, MAP and HR did not differ between conditions in the overweight/obese group. In the normal-weight group, TAG across the 5-h was attenuated by -0.12 mmol^.^L^−1^ (95% CI -0.22, -0.03) in INT-SIT than SIT (Table 2). There were no differences between conditions for the remaining cardiometabolic risk markers in the normal-weight group. There were no condition x time interaction effects for any variable in either group (see Fig. 2 and Supplementary Material S3; all *p* ≥ 0.27).

### Substrate utilisation

Fat and carbohydrate oxidation across the 5-h were higher in INT-SIT by 2.81 mg^.^min^−1^ (95% CI 0.52, 5.09) and 11.41 mg^.^min^−1^ (95% CI 4.40, 18.43), respectively, compared with SIT in the overweight/obese group (see Supplementary Material S4). Postprandial substrate utilisation iAUC did not differ between conditions in the overweight/obese group. Resting energy expenditure across 5-h and iAUC were higher by 0.04 kcal^.^min^−1^ (95% CI 0.02, 0.06) and 0.05 kcal^.^min^−1^ (95% CI 0.00, 0.09), respectively, in INT-SIT than SIT in the overweight/obese group (see Supplementary Material S4). There was no condition x time interaction for any substrate utilisation outcomes in the overweight/obese group (*p* ≥ 0.81). In the normal-weight group, the main effect of condition and condition x time interactions for substrate utilisation outcomes were all non-significant (*p* ≥ 0.12) (see Supplementary Material S5).

## Discussion

The findings of this study supported the hypothesis that interrupting sitting with 5-min bouts of light walking every 30-min acutely attenuated postprandial glucose, TAG and MLI in South Asians with overweight/obesity, with limited effects seen in participants with normal-weight. This supports previous research that has consistently found beneficial cardiometabolic responses to interrupting sitting with 2 to 5-min of walking every 20 to 30-min in overweight/obese Caucasians (Dunstan et al. 2012; Henson et al. 2016; Larsen et al. 2015). Similar to the present findings, other studies have found no changes in postprandial glucose or TAG when sitting was interrupted with light or moderate-intensity walking or cycling for 2 to 8-min every 20 to 60-min in normal-weight Caucasians (Altenburg et al. 2013; Hansen et al. 2016), although the literature is conflicting (Bailey and Locke 2015; Bailey et al. 2017; Peddie et al. 2013). This evidence suggests that interrupted sitting may provide a more pronounced and consistent cardiometabolic effect in participants with overweight or obesity than normal-weight individuals, perhaps due to their larger ‘scope for improvement’. Indeed, previous research shown that interrupted sitting with light walking consistently attenuates cardiometabolic risk markers (e.g., glucose) in populations with increased cardiometabolic risk, including individuals with overweight/obesity, Type 2 diabetes, and postmenopausal women (Dempsey et al. 2016; Dunstan et al. 2012; Henson et al. 2016; Larsen et al. 2015). The magnitude of response also appears to be comparatively greater in these population groups than in apparently healthy individuals (Hansen et al. 2016). The findings of the current study support a focus on interrupting sitting for managing cardiometabolic health in the short term, especially in individuals with overweight/obesity, regardless of their ethnicity.

There was a trend for interrupting sitting with light walking to attenuate postprandial glucose area under the curve (by 13% for iAUC) in South Asians with overweight/obesity, whereas no such trend was apparent for postprandial insulin. In Caucasians living with overweight/obesity, interrupting sitting with light walking for 2 to 5-min every 20 to 30-min attenuated postprandial glucose iAUC by 28% to 32% (Henson et al. 2016; Larsen et al. 2015) and insulin iAUC by 15% to 37% (Dunstan et al. 2012; Henson et al. 2016). The greater responses in these previous studies may have reflected the participants having larger impairments in cardiometabolic health (e.g., reduced glucose tolerance) and lower aerobic fitness than the present study’s sample. This cannot be said conclusively, though, as such measures of physical and cardiometabolic health are not taken consistently across studies. In Caucasians with normal-weight, reductions of 16% and 21% in glucose and insulin iAUC, respectively, have been found when sitting is interrupted with 2-min light walking every 20-min (Bailey and Locke 2015; Pulsford et al. 2017). The magnitude of the glucose reduction in the present study is comparatively lower. In addition to potential explanatory factors discussed above, the less frequent interruptions in sitting (i.e., every 30-min) compared with previous studies (i.e., every 20-min) could account for the lack of change in postprandial metabolism. Further studies are, therefore, required to understand the cardiometabolic effects of interrupting sitting with different frequencies and durations of physical activity in South Asians to better inform recommendations for managing cardiometabolic health. This research should also investigate the influence of physical and cardiometabolic health on postprandial metabolism.

In the only previous experimental study with South Asian participants (older adults aged > 65 years with a BMI of 26 [95% CI 23.7, 29.5] kg.m^−2^), interrupting sitting with light walking for 5-min every 30-min reduced postprandial insulin AUC by 27%, but did not affect postprandial glucose (Yates et al. 2020). The attenuated glucose in the absence of insulin changes in the present study may reflect an improved insulin sensitivity, or enhanced contraction-mediated glucose uptake, in response to interrupting sitting. In the study by Yates et al. (2020), participants were comparatively older (mean age 69 years) compared to the present participants (mean age 50 years). It is possible that postprandial glucose and insulin responses could be different in younger individuals due to differences in muscle or adipose tissue physiology, or fitness level (Bowden et al. 2019). The longer experimental condition in the study by Yates et al. (2020) meant that more walking bouts were accumulated that could have provided a greater stimulus for glucose uptake across the day, resulting in lower concentrations of insulin being required. The higher percentage of carbohydrate in the test meals in the present study could have resulted in a higher postprandial glucose response with more scope for reductions through interrupting sitting. These data suggest that interrupting sitting with light walking benefits postprandial metabolism in South Asian adults with overweight/obesity. Whether this is in the context of attenuated glucose or insulin may depend on the characteristics of the participants, the number of sitting interruptions or the test meal composition. It is recommended that interrupting sitting with light walking be considered as a potential strategy for improving postprandial metabolism in this ethnic group.

The present study observed reductions in postprandial TAG in both the overweight/obese and normal-weight groups in response to interrupting sitting. This is similar to studies in which sitting was interrupted with 3-min walking bouts in participants with normal-weight or overweight/obesity (Miyashita et al. 2015, 2006). Metabolic load index, which is a summative index reflecting excess energy from glucose and triglycerides, was reduced in the interrupted sitting condition in South Asian participants with overweight/obesity, but not in those with a normal-weight. Higher adiposity, that is associated with metabolic inflexibility and insulin resistance, may explain the more pronounced effects in individuals with overweight/obesity (Bays et al. 2022; Sattar and Gill 2015). Previous studies have not evaluated MLI. It is recommended that this outcome be considered in future research to extend knowledge regarding cardiometabolic responses to interrupting sitting.

Consistent with studies in normal-weight Caucasians (Charlett et al. 2021) and Caucasians living with overweight/obesity (Larsen et al. 2014), the present study found no change in MAP and HR in response to interrupting sitting. In contrast, other research has reported reductions in MAP (Barone Gibbs et al. 2017) when alternating between sitting and standing every 30-min in individuals with overweight/obesity and hypertension. It is plausible that individuals with hypertension may respond to interruptions in sitting to a greater extent than individuals with normal blood pressure levels. Alternatively, sitting may need to be interrupted for a longer duration for MAP benefits to be realised. This is supported with evidence of reduced MAP in response to alternating sitting and standing every 30-min (Barone Gibbs et al. 2017) and light walking for 20-min bouts each hour (Champion et al. 2018). According to the findings presented here, interrupting sitting with 5-min bouts of light walking every 30-min does not benefit MAP, acutely, in South Asians. Informed by previous data in Caucasians (Barone Gibbs et al. 2017; Champion et al. 2018), the effects of interrupting sitting with longer bouts of standing or physical activity should be examined in this ethnic group.

There was an increase in REE by 25% in response to interrupted sitting with light walking in South Asians living with overweight/obesity. Resting energy expenditure was also increased by up to 20% when interrupting sitting with chair squats (standing and sitting 10 times for 30-s every 20-min) or intermittent standing (standing 10 times for 1.5-min every 30-min) compared to prolonged sitting in adults Caucasian living with overweight/obesity (Hawari et al. 2019, 2016). The higher REE may be explained by higher fat (+ 14%) and carbohydrate (+ 19%) oxidation in response to interrupting sitting with light walking in the present study. Increased carbohydrate and fat oxidation were also reported in previous studies using chair squats, intermittent standing, and light or moderate-intensity walking bouts (Hawari et al. 2019, 2016; Peddie et al. 2013). The higher REE and substrate oxidation would aid in replenishing the additional energy used for muscle contractions during the sitting interruptions. Despite these effects in the overweight/obese group, the present study did not observe any changes in the normal-weight group. The benefits of interrupted sitting could be more pronounced in individuals with overweight/obesity compared with normal-weight due to walking being a weight-bearing activity, meaning a greater energy demand for those carrying more weight, or a lower fitness resulting in higher metabolic stress. That said, the 0.06 kcal/min^−1^ higher REE compared with prolonged sitting would equate to an additional energy expenditure of 126 kcal per week, which could have limited implications for long-term weight management. The magnitude of effect on substrate utilisation in South Asians with overweight/obesity should be explored in future studies with differing protocols for interrupting sitting to inform the potential of this strategy for weight management.

### Strengths and limitations

This study adds important knowledge to the scarce literature investigating the effects of interrupted sitting on cardiometabolic risk markers and resting substrate utilisation in South Asians living with normal-weight and overweight/obesity. The strengths of this study also include the randomised cross-over design in a controlled laboratory setting, where manipulations in sitting and dietary intake were strictly controlled throughout the experimental protocol. This acute study meant that any long-term effects were not examined. A further limitation is the evaluation of postprandial responses to standardised meals that consisted of foods that may not reflect usual dietary intake in South Asians; thus, the findings may lack ecological validity. The COVID-19 pandemic also caused data collection to be suspended, meaning that there were not sufficient participants to examine a condition x weight status interaction. This should be explored in the future to provide definitive conclusions regarding the effects of interrupting sitting across South Asians of differing weight status. Finally, the cardiometabolic health benefits of interrupting sitting were not directly compared to other ethnic groups (e.g., Caucasians). Thus, future research should investigate the interacting effects of interrupting sitting in participants with normal-weight and overweight/obesity across different ethnic groups.

## Conclusion

Interrupted sitting with 5-min bouts of light walking every 30-min acutely attenuated postprandial glucose, TAG and MLI in South Asians with overweight/obesity. In normal-weight South Asians, there was less widespread cardiometabolic health benefits from this type of acute intervention. Resting energy expenditure from both carbohydrate and fat oxidation was increased with light walking bouts only in participants living with overweight/obesity. Future research should examine the effectiveness of interrupting sitting in free-living settings and over the longer term in South Asians to inform potential public health intervention targets for this ethnic group.

### Supplementary Information

Below is the link to the electronic supplementary material.

Supplementary file1 (DOCX 30 KB)

Supplementary file2 (PNG 163 KB)

Supplementary file3 (PNG 1883 KB)

Supplementary file4 (DOCX 26 KB)

Supplementary file5 (PNG 2203 KB)

## Data Availability

The datasets generated during and/or analysed during the current study are available from the corresponding author on reasonable request.

## Abbreviations

BMI: Body mass index
SIT: Prolonged sitting
INT-SIT: Interrupted sitting
iAUC: Incremental area under the curve
REE: Resting energy expenditure
TAG: Triglycerides
MLI: Metabolic load index
CVD: Cardiovascular disease
CONSORT: Consolidated Standards of Reporting Trials
BP: Blood pressure
HR: Heart rate
RPE: Borg rating of perceived exertion
GI: Glycaemic index
tAUC: Total area under the curve
p-iAUC: Positive incremental area under the curve
MAP: Mean arterial pressure
CI: Confidential interval
