# Supplementary figures and images for: Interrupting sitting acutely attenuates cardiometabolic risk markers in South Asian adults living with overweight and obesity
Source: Eur J Appl Physiol. 2023 Nov 11;124(4):1163–74. doi: 10.1007/s00421-023-05345-7 (PMC10954978; doi:10.1007/s00421-023-05345-7)

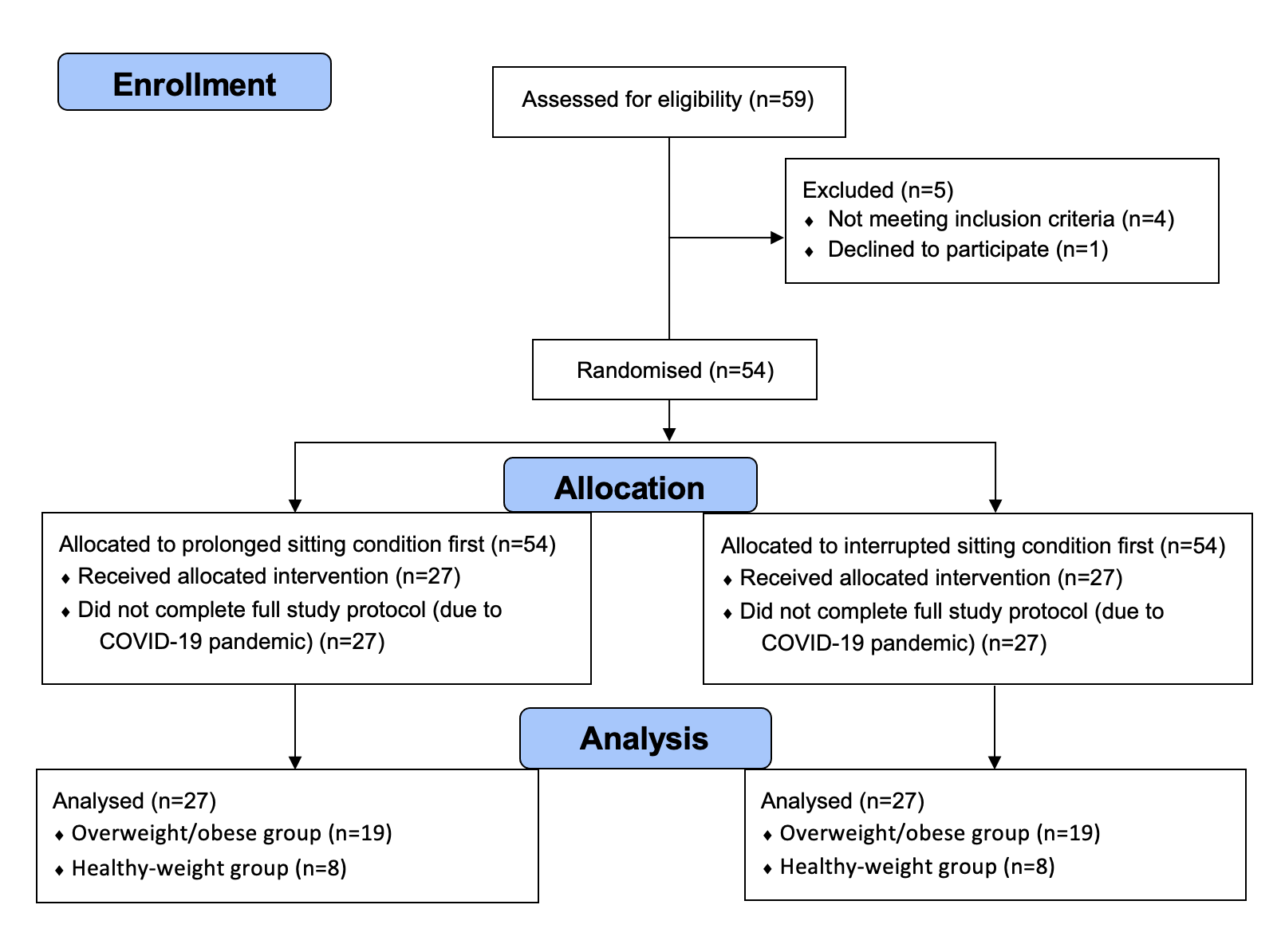

Supplement: Supplementary file 2 — Supplementary file2 (PNG 163 KB) [file 421_2023_5345_MOESM2_ESM.png]

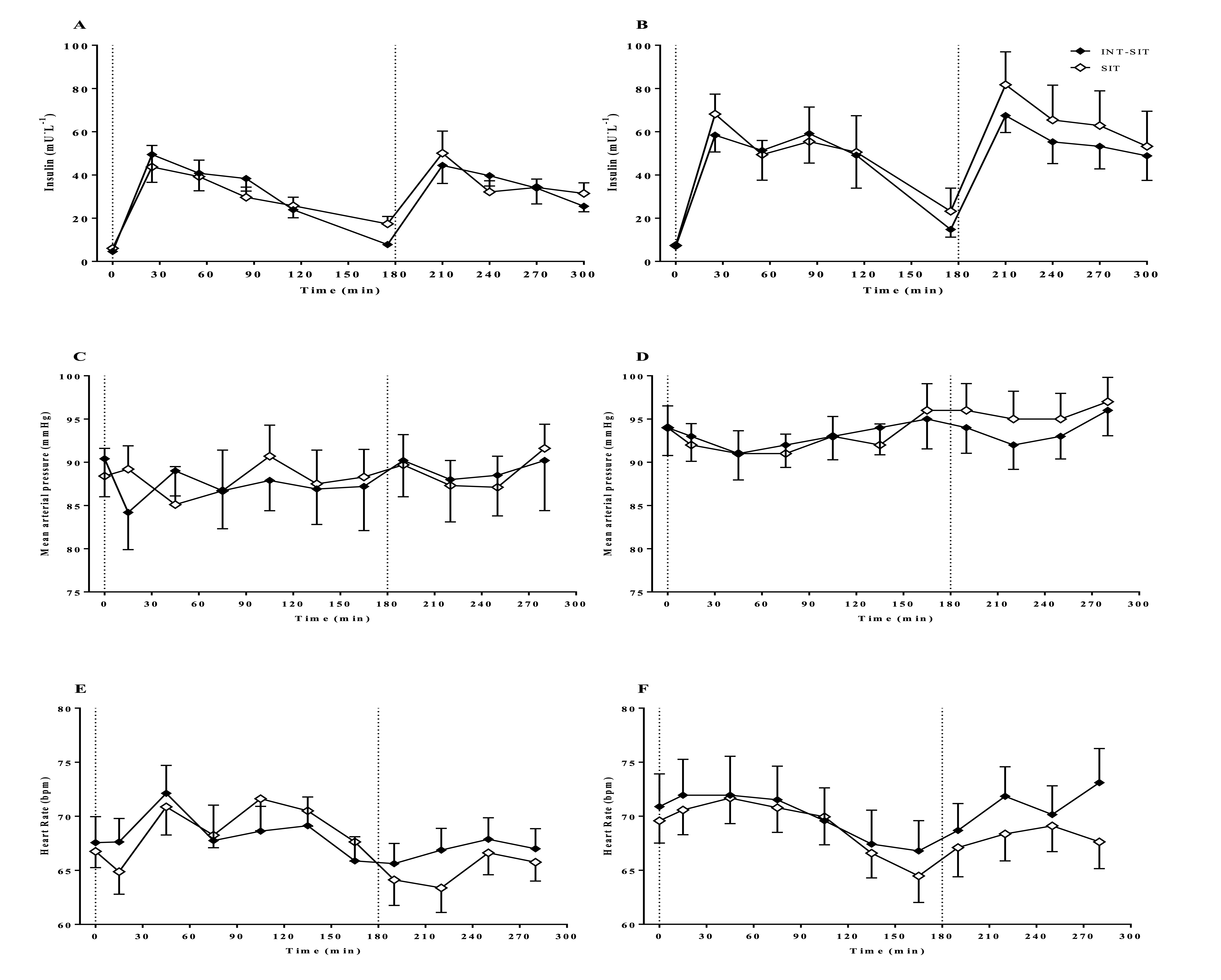

Supplement: Supplementary file 3 — Supplementary file3 (PNG 1883 KB) [file 421_2023_5345_MOESM3_ESM.png]

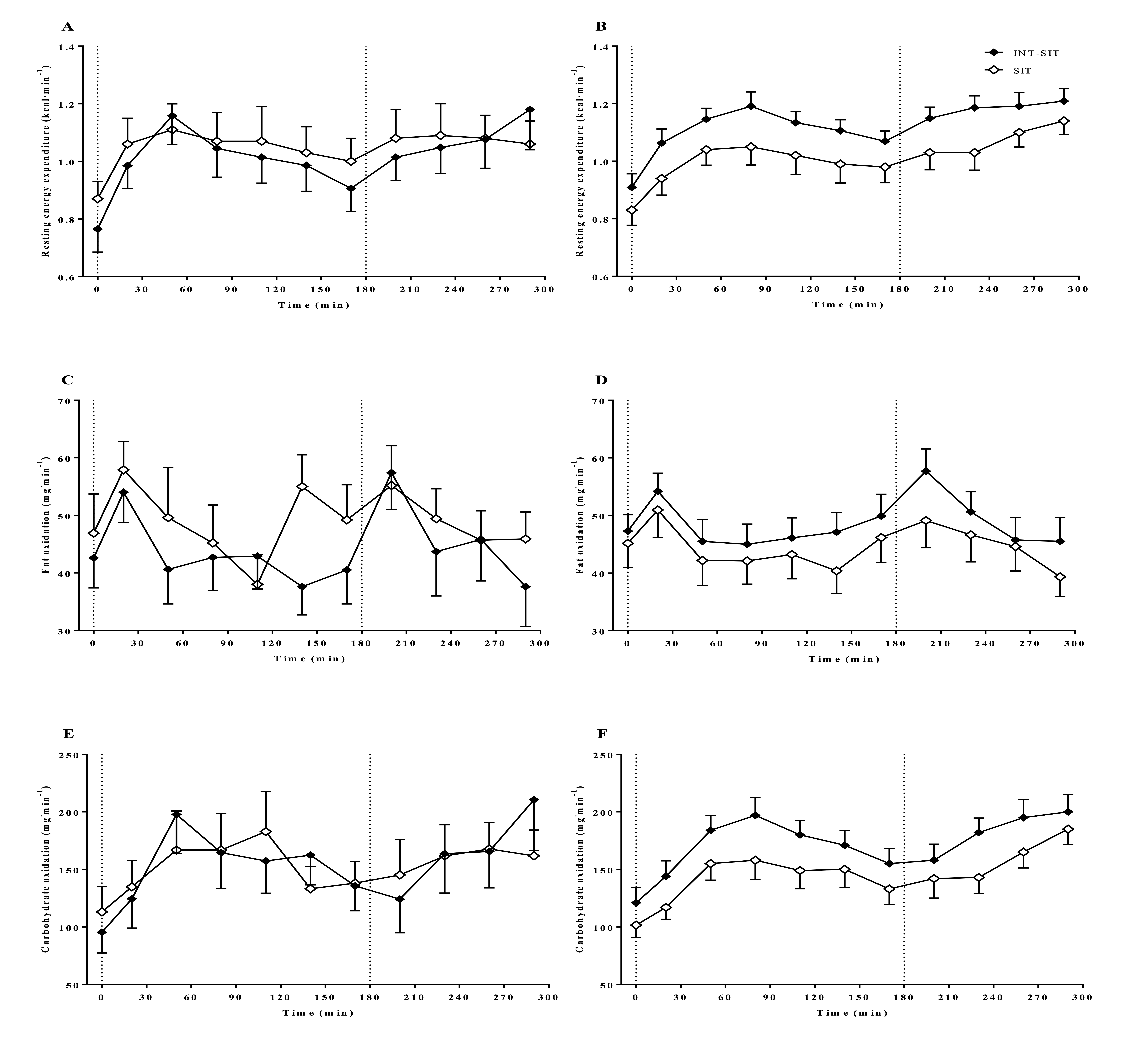

Supplement: Supplementary file 5 — Supplementary file5 (PNG 2203 KB) [file 421_2023_5345_MOESM5_ESM.png]
