# Supplementary material for: Interrupting sitting acutely attenuates cardiometabolic risk markers in South Asian adults living with overweight and obesity
Source: Eur J Appl Physiol. 2023 Nov 11;124(4):1163–74. doi: 10.1007/s00421-023-05345-7 (PMC10954978; doi:10.1007/s00421-023-05345-7)
Supplement: Supplementary file 4 — Supplementary file4 (DOCX 26 KB) [file 421_2023_5345_MOESM4_ESM.docx]

Supplementary Material S4. Resting substrate utilisation and energy expenditure in the two experimental conditions.

|  | Prolonged sitting | Interrupted sitting with light walking | *p*-value for the main effect of condition | Cohens’ *d*  effect size |
| --- | --- | --- | --- | --- |
| Overweight/obese participants (n=19) |  |  |  |  |
| Baseline resting fat oxidation (mg^.^min^-1^) | 43.81 (37.04, 50.59) | 45.91 (39.14, 52.69) | 0.66 | 0.13 |
| Baseline resting carbohydrate oxidation (mg^.^min^-1^) | 103.95 (79.87, 128.02) | 123.36 (99.28, 147.43) | 0.22 | 0.36 |
| Baseline resting energy expenditure (kcal^.^min^-1^) | 0.82 (0.72, 0.93) | 0.91 (0.80, 1.01) | 0.31 | 0.37 |
| Resting fat oxidation tAUC (mg^.^min^-1^) | 44.22 (38.47, 49.96) | 46.99 (41.28, 52.71) | 0.48 | 0.23 |
| Resting fat oxidation iAUC (mg^.^min^-1^) | 2.42 (-3.33, 8.18) | 2.12 (-3.62, 7.85) | 0.91 | 0.13 |
| Resting fat oxidation over time^a^ (mg^.^min^-1^) | 45.57 (41.21, 49.94) | 48.38 (44.02, 52.74) | **0.02** | 0.23 |
| Resting carbohydrate oxidation tAUC (mg^.^min^-1^) | 154.78 (136.60, 172.95) | 160.36 (142.02, 178.69) | 0.54 | 0.14 |
| Resting carbohydrate oxidation iAUC (mg^.^min^-1^) | 43.57 (26.53, 60.61) | 52.38 (35.20, 69.57) | 0.31 | 0.24 |
| Resting carbohydrate oxidation over time^a^ (mg^.^min^-1^) | 151.62 (136.48, 166.77) | 163.04 (147.86, 178.22) | **0.001** | 0.48 |
| Resting energy expenditure tAUC (kcal^.^min^-1^) | 1.02 (0.97, 1.06) | 1.06 (1.02, 1.11) | **0.04** | 0.51 |
| Resting energy expenditure iAUC (kcal^.^min^-1^) | 0.18 (0.13, 0.22) | 0.23 (0.18, 0.27) | **0.04** | 0.51 |
| Resting energy expenditure over time^a^ (kcal^.^min^-1^) | 1.05 (1.01, 1.09) | 1.09 (1.05, 1.13) | **<0.001** | 0.53 |

|  | Prolonged sitting | Interrupted sitting with light walking | *p*-value for the main effect of condition | Cohens’ *d*  effect size |
| --- | --- | --- | --- | --- |
| Normal-weight participants (n=8) |  |  |  |  |
| Baseline resting fat oxidation (mg^.^min^-1^) | 46.45 (32.04, 60.86) | 42.18 (27.77, 56.60) | 0.71 | 0.25 |
| Baseline resting carbohydrate oxidation (mg^.^min^-1^) | 109.21 (58.86, 159.56) | 91.59 (41.23, 141.94) | 0.50 | 0.31 |
| Baseline resting energy expenditure (kcal^.^min^-1^) | 0.86 (0.63, 1.08) | 0.75 (0.52, 0.97) | 0.07 | 0.42 |
| Resting fat oxidation tAUC (mg^.^min^-1^) | 46.43 (36.87, 55.98) | 43.91 (34.36, 53.46) | 0.63 | 0.20 |
| Resting fat oxidation iAUC (mg^.^min^-1^) | 3.21 (-6.35, 12.76) | 0.67 (-8.89, 10.23) | 0.63 | 0.21 |
| Resting fat oxidation over time^a^ (mg^.^min^-1^) | 47.66 (36.53, 58.79) | 45.44 (34.31, 56.57) | 0.26 | 0.35 |
| Resting carbohydrate oxidation tAUC (mg^.^min^-1^) | 144.99 (93.68, 196.30) | 155.46 (104.15, 206.77) | 0.61 | 0.16 |
| Resting carbohydrate oxidation iAUC (mg^.^min^-1^) | 44.28 (-7.05, 95.61) | 54.75 (3.43, 106.08) | 0.61 | 0.16 |
| Resting carbohydrate oxidation over time^a^ (mg^.^min^-1^) | 148.01 (86.81, 209.22) | 159.75 (98.52, 220.98) | 0.12 | 0.01 |
| Resting energy expenditure tAUC (kcal^.^min^-1^) | 1.01 (0.81, 1.21) | 1.00 (0.80, 1.20) | 0.84 | 0.05 |
| Resting energy expenditure iAUC (kcal^.^min^-1^) | 0.22 [0.02, 0.42) | 0.21 [0.01, 0.41) | 0.82 | 0.05 |
| Resting energy expenditure over time^a^ (kcal^.^min^-1^) | 1.03 (0.77, 1.29) | 1.00 (0.53, 1.42) | 0.17 | 0.13 |

Data presented as mean (95% confidence interval); tAUC, total area under the curve; iAUC, incremental area under the curve; ^a^values refer to the marginal means for the main effect of condition in the condition by time analyses; Statistically significant (*p*≤0.05) differences highlighted in **bold.**
